# Skin microbiome before development of atopic dermatitis: Early colonization with commensal staphylococci at 2 months is associated with a lower risk of atopic dermatitis at 1 year

**DOI:** 10.1016/j.jaci.2016.07.029

**Published:** 2017-01

**Authors:** Elizabeth A. Kennedy, Jennifer Connolly, Jonathan O'B. Hourihane, Padraic G. Fallon, W.H. Irwin McLean, Deirdre Murray, Jay-Hyun Jo, Julia A. Segre, Heidi H. Kong, Alan D. Irvine

**Affiliations:** a Dermatology Branch, Center for Cancer Research, National Cancer Institute, National Institutes of Health, Bethesda, Md; b Paediatrics and Child Health, University College, Cork, and the Irish Centre for Fetal and Neonatal Translational (INFANT) Research, Cork University Maternity Hospital, Cork, Ireland; c Clinical Medicine, Trinity College Dublin, Dublin, Ireland; d National Children's Research Centre, Our Lady's Children's Hospital Crumlin, Dublin, Ireland; g Paediatric Dermatology, Our Lady's Children's Hospital Crumlin, Dublin, Ireland; e Dermatology and Genetic Medicine, University of Dundee, Dundee, United Kingdom; f Translational and Functional Genomics Branch, National Human Genome Research Institute, National Institutes of Health, Bethesda, Md

**Keywords:** *Staphylococcus aureus*, atopic dermatitis, skin, microbiome, longitudinal birth cohort, 16S sequencing, AD, Atopic dermatitis, Af, Antecubital fossa, AMOVA, Analysis of molecular variance, BASELINE, Babies After SCOPE: Evaluating the Longitudinal Impact Using Neurological and Nutritional Endpoints, FLG, Filaggrin, Nt, Nasal tip, OTU, Operational taxonomic unit, Pf, Popliteal fossa

## Abstract

**Background:**

Disease flares of established atopic dermatitis (AD) are generally associated with a low-diversity skin microbiota and *Staphylococcus aureus* dominance. The temporal transition of the skin microbiome between early infancy and the dysbiosis of established AD is unknown.

**Methods:**

We randomly selected 50 children from the Cork Babies After SCOPE: Evaluating the Longitudinal Impact Using Neurological and Nutritional Endpoints (BASELINE) longitudinal birth cohort for microbiome sampling at 3 points in the first 6 months of life at 4 skin sites relevant to AD: the antecubital and popliteal fossae, nasal tip, and cheek. We identified 10 infants with AD and compared them with 10 randomly selected control infants with no AD. We performed bacterial 16S ribosomal RNA sequencing and analysis directly from clinical samples.

**Results:**

Bacterial community structures and diversity shifted over time, suggesting that age strongly affects the skin microbiome in infants. Unlike established AD, these patients with infantile AD did not have noticeably dysbiotic communities before or with disease and were not colonized by *S aureus*. In comparing patients and control subjects, infants who had affected skin at month 12 had statistically significant differences in bacterial communities on the antecubital fossa at month 2 compared with infants who were unaffected at month 12. In particular, commensal staphylococci were significantly less abundant in infants affected at month 12, suggesting that this genus might protect against the later development of AD.

**Conclusions:**

This study suggests that 12-month-old infants with AD were not colonized with *S aureus* before having AD. Additional studies are needed to confirm whether colonization with commensal staphylococci modulates skin immunity and attenuates development of AD.

*Discuss this article on the JACI Journal Club blog:* *www.jaci-online.blogspot.com*.

Atopic dermatitis (AD) is a common inflammatory skin condition that begins early in life. Patients with AD with established disease experience frequent colonization and increased infections with *Staphylococcus aureus*, as well as potentially life-threatening eczema herpeticum with herpes simplex virus. The hygiene hypothesis relates the development of atopic disorders (AD, allergic rhinitis, and asthma) to reduced microbial exposure at a young age.^1^ Epidemiologic studies examining the incidence of asthma have linked exposure to farming environments to lower rates of allergic disorders.^2^, ^3^, ^4^ However, the potential role of microbe exposure in early childhood to the development of AD and the subsequent atopic march toward the development of allergic rhinitis and asthma remains to be elucidated.

There is significant interest in the potential effects of microbes on the development of skin immunity, as well as disease.^5^, ^6^, ^7^, ^8^, ^9^ Recent work in mice has shown that cutaneous exposure to commensal bacteria early in life can induce tolerance to these microbes.^6^ Given these epidemiologic associations between environmental exposure and development of atopic diseases, we investigated the skin microbiome in a birth cohort. We analyzed bacterial 16S rRNA gene sequences from swabs collected from 4 skin sites in infants in a birth cohort (Babies After SCOPE: Evaluating the Longitudinal Impact Using Neurological and Nutritional Endpoints [BASELINE]) at 3 different time points to determine whether differences in the skin microbiome were associated with AD development.

## Methods

### Study subjects

The Cork BASELINE Birth Cohort Study is the pediatric follow-on from the Cork Centre for the Screening for Pregnancy Endpoints (SCOPE) study.^10^, ^11^ The Cork BASELINE Birth Cohort Study recruited within a white Irish population in Cork, Ireland, from August 2009 to October 2011. These women were subject to the inclusion criteria of the SCOPE study: low-risk primigravidous mothers with singleton pregnancies who delivered at or near term. Maternal consent was obtained at 20 weeks' gestation and verified at delivery. Ethical approval for the Cork BASELINE Birth Cohort Study was granted by the Clinical Research Ethics Committee of the Cork Teaching Hospitals (ref ECM 5 [9] 01/07/2008). The BASELINE study is registered with the US National Institutes of Health Clinical Trials Registry (http://www.clinical trials.gov; ID: NCT01498965).

### Clinical diagnosis of AD

All infants were assessed at birth and 2, 6, 12, and 24 months of age. Assessment included parental questionnaires and physical examination. Screening questions specific for AD were included in the questionnaires administered at 2, 6, and 12 months. AD was diagnosed (at 6, 12, and 24 months) by experienced health care personnel using the UK Working Party Diagnostic Criteria.^12^, ^13^, ^14^ When AD was present, the SCORAD clinical tool was used to assess severity.^15^, ^16^ The Nottingham Eczema Severity Score was also used to assess AD severity at 12 months.^17^ Demographic data and clinical details are shown in Table I and Table E1 in this article's Online Repository at www.jacionline.org.

### Filaggrin genotyping

Cord blood samples were collected at birth and stored for analysis. Filaggrin *(FLG)* genotyping was carried out on all study subjects with testing for the 4 most common Irish/European mutations, as previously described.^18^ None of the subjects in this study were found to have *FLG* mutations.

### Sampling for microbiome analysis

We randomly selected 50 infants from the birth cohort and obtained skin swabs at day 2, month 2, and month 6. Skin samples and negative controls were collected with premoistened swabs, as previously described.^19^ After all infants had been assessed at 1 year, 10 infants with clinical AD at months 2, 6, and/or 12 were selected for analysis as patients with AD. Healthy control subjects were 10 infants without AD at any study time points selected at random. Sample sites were selected based on the presentation of AD at different ages. Cheeks are a site of AD predilection in infants, and the nasal tip (Nt) is typically spared. The antecubital fossa (Af) and popliteal fossa (Pf) are typical sites of AD predilection in children and adolescents.

### Sample processing/sequencing

16S rRNA V1-V3 sequencing was performed on swab samples, as previously described.^19^ Swabs were incubated in Yeast Cell Lysis Solution (MasterPure Kit, MPY80200; Epicentre, Madison, Wis) and Ready-Lyse Solution (R1802M, Epicentre) for 1 hour at 37°C. Two 5-mm stainless steel beads (Qiagen, Hilden, Germany) were added and processed in a TissueLyser (Qiagen) for 2 minutes at 30 Hz. The solution was treated with MPC Protein Precipitation Reagent (MasterPure Kit MPY80200, Epicentre) to remove cellular debris. Subsequent steps were performed with the PureLink Genomic DNA Kit (Invitrogen, Carlsbad, Calif). Barcoded primers flanking V1 (27F, 59-AGAGTTTGATCCTGGCTCAG-39) and V3 (534R, 59-ATTACCGCGGCTGCTGG-39) were used for PCR. PCR products were purified with the Agencourt AMPure XP Kit (A63880; Beckman Coulter, Brea, Calif) and quantitated with the Quant-iT dsDNA High-Sensitivity Assay Kit (Q33120, Invitrogen); equivalent amounts of these PCR products were pooled, purified with a Qiagen MinElute column (28004, Qiagen) into 30 μL of TE buffer (10 mM Tris·Cl, 1 mM EDTA, pH 8.0), and sequenced at the National Institutes of Health Intramural Sequencing Center on a 454 GS FLX (Roche, Mannheim, Germany) platform. Reagents and collection procedure controls were tested and demonstrated no significant background contamination.

### Data analysis

**Fig E1:**
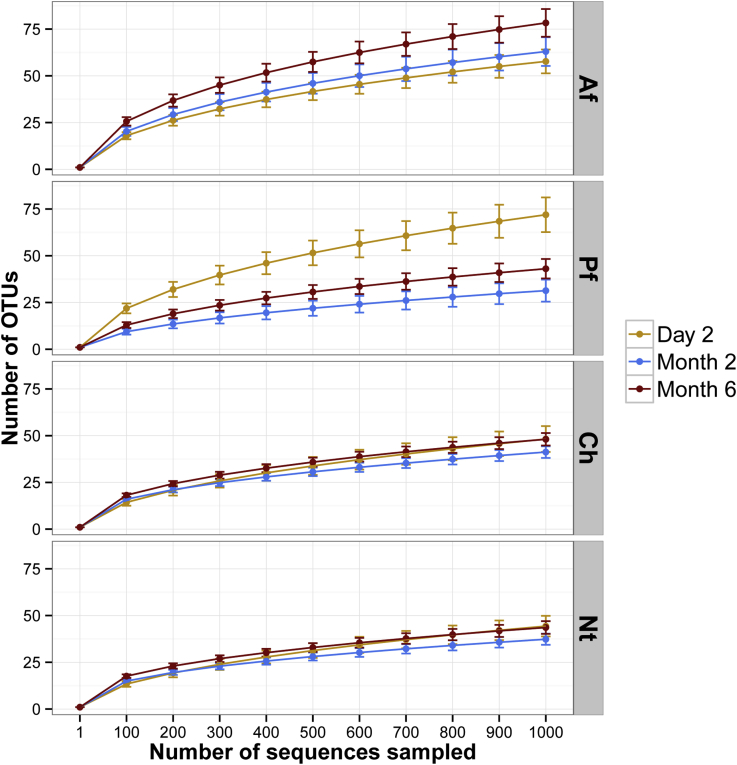


Sequences were preprocessed with mothur version 1.35.1.^20^ Briefly, 454 flowgram data were trimmed and denoised, and chimera checking was completed with the mothur implementation of UCHIME.^21^ Sequences were classified by using the Ribosomal Database Project naive Bayesian classifier.^22^ Sequences classified as chloroplast or mitochondria were discarded. Site-specific definition of operational taxonomic units (OTUs; groups of sequences that share a specific level of similarity) and downstream analyses was performed in mothur. Within the samples from each time point or site, pairwise distances were calculated, and OTUs were defined at 97% nucleotide similarity. Within-sample (Shannon diversity) and between-sample (theta index) measurements were performed based on these OTU definitions, with subsampling to 1000 sequences per sample.^23^ Rarefaction curves level off by this value, suggesting adequate sequencing coverage; any samples with fewer than 1000 sequences after preprocessing were removed from analysis (see Fig E1 in this article's Online Repository at www.jacionline.org). Differentially abundant OTUs were detected by using the metastats command in mothur.

The sequences classified to the *Staphylococcus* genus by using the RDP naive Bayesian classifier were then placed on a phylogenetic reference tree using “-keep-at-most 1000 max-pitches 1000.” Taxonomy was assigned by using the guppy program in pplacer,^24^ with a likelihood cutoff set to 0.65, as previously described.^19^

### Statistics

All data analysis was performed in R software; results are presented as means ± SEMs, unless otherwise indicated. Ninety-five percent CIs were estimated. *Post hoc* tests (ie, pairwise comparisons in analysis of molecular variance [AMOVA]) were adjusted by using Bonferroni correction. For detection of differentially abundant OTUs, Metastats results are filtered for OTUs with a mean abundance of 0.05% or greater, and *P* values were calculated by using a false discovery rate adjustment.

## Results

### Site-specific bacterial colonization patterns

**Fig E2:**
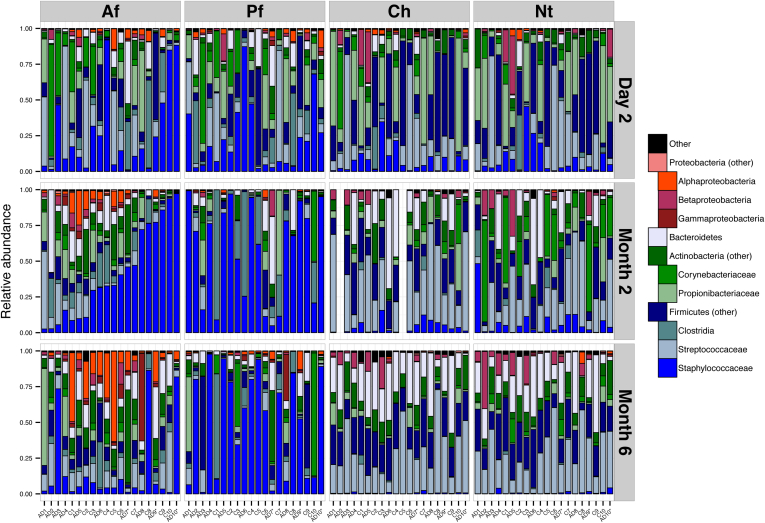


Because different skin microenvironments and anatomic regions harbor distinct microbial communities in adults and older children,^25^, ^26^ we initially compared the major bacterial taxa present at the 4 sites on infants. Relative abundances of these bacterial taxa showed differences between the 2 facial sites (cheeks and Nt) and the extremity sites (Af and Pf, see Fig E2 in this article's Online Repository at www.jacionline.org). Calculation of differentially abundant species between the site types confirmed that *Staphylococcus* species were relatively more abundant on extremity sites at all time points and *Gemella* species were relatively more abundant on facial sites. Other taxa were only differentially represented at some time points, with facial sites higher in *Propionibacterium* species at day 2 and *Streptococcus* species at later time points (see Table E2 in this article's Online Repository at www.jacionline.org).

**Fig E3:**
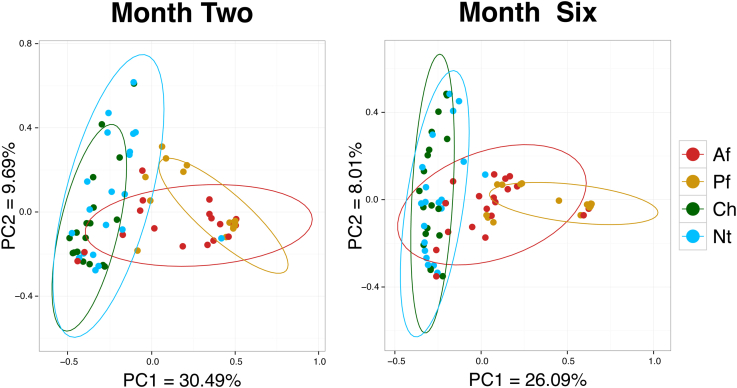


**Fig 1:**
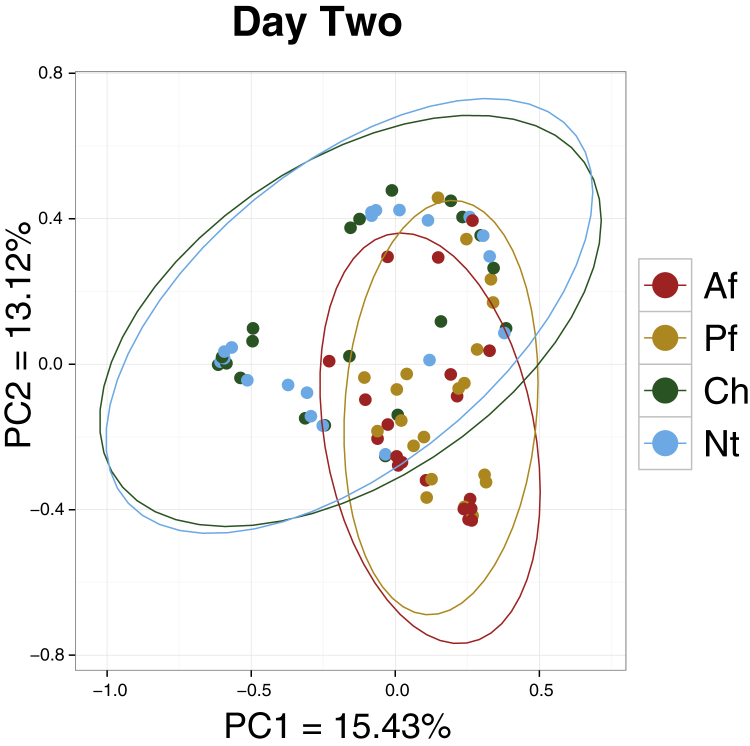
Site specificity of bacterial community composition. All samples at day 2 were clustered by using principal coordinates analysis based on theta similarity coefficients. At day 2, Af and Pf clustered together (*P* = 1, AMOVA), as did cheeks *(Ch)* and Nt (*P* = 1), but each clustered distinctly from the other site pair (*P* < .006). *P* values were adjusted with Bonferroni correction (n = 6).

We validated these findings with biodiversity calculations, examining samples from each time point. We analyzed how similar the bacterial community structures were between the samples using the theta similarity index, which accounts for both the presence and proportion of bacterial species.^23^ A theta index value of 1 indicates that the 2 bacterial communities have identical structures; a value of 0 indicates maximal dissimilarity. In principal coordinates analyses based on these theta values, samples that are more similar to each other cluster more closely together. At each time point, the facial site samples clustered together (*P* > .05, AMOVA) but are distinctly separate from the extremity site samples (*P* < .006). The extremity sites clustered together at day 2 and month 2 but had different centroids at month 6 (*P* ≤ .006, Fig 1 and see Fig E3 in this article's Online Repository at www.jacionline.org).

### Changes in bacterial colonization over time

**Fig E4:**
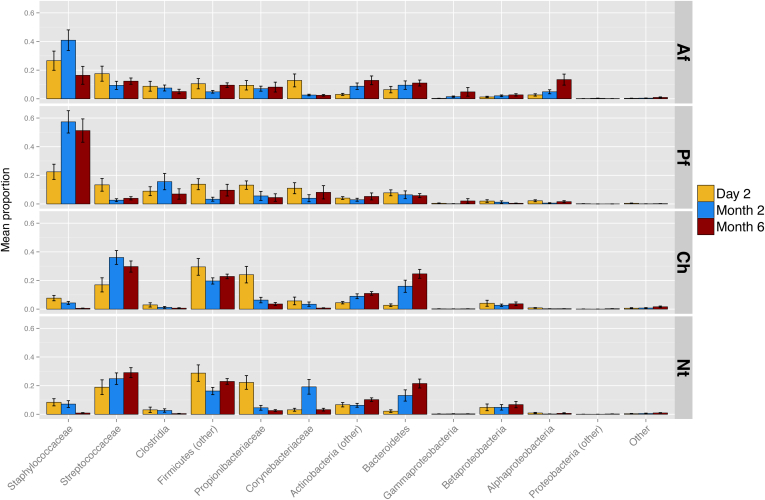


**Fig E5:**
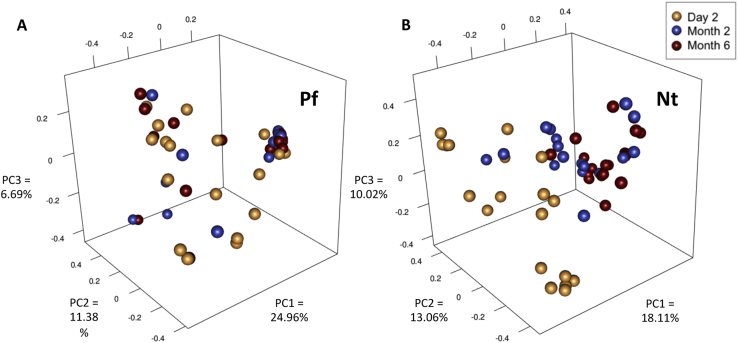


**Fig 2:**
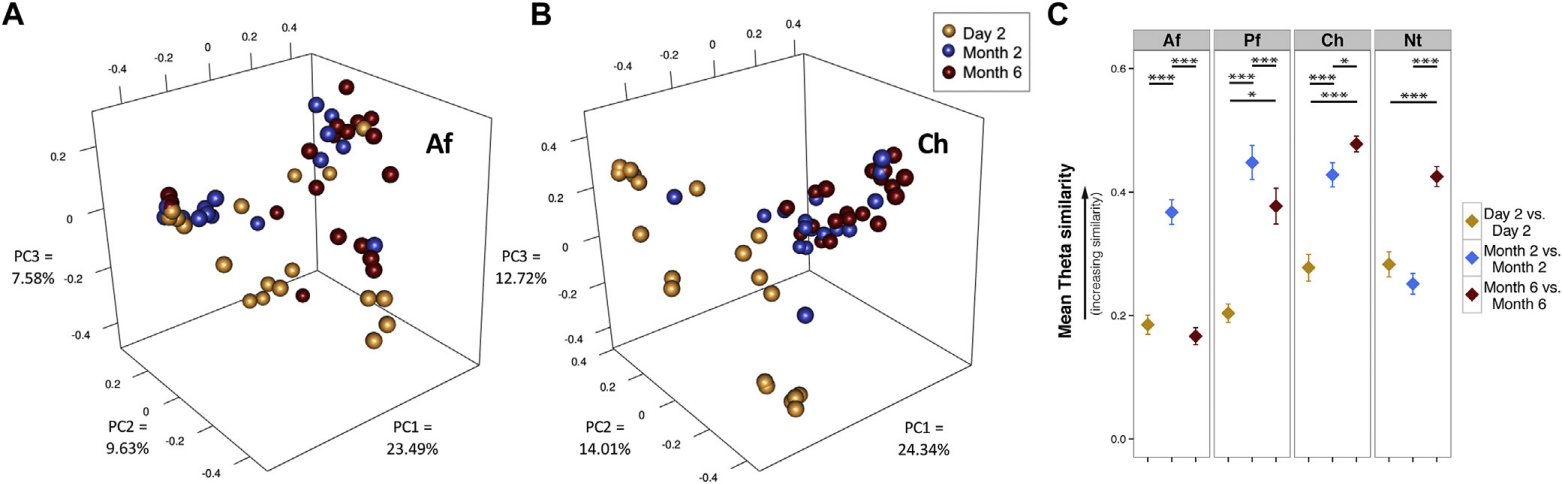
Skin microbial communities in infants undergo site-specific shifts in composition with age. **A,** Af samples clustered by using principal coordinates analysis based on theta similarity coefficients. By using AMOVA, samples clustered distinctly between day 2 and month 6 (*P* = .024) but not significantly between month 2 and month 6 (*P* = .12) or between day 2 and month 2 (*P* = .21). **B,** Cheek samples clustered by using principal coordinates analysis based on theta similarity coefficients. By using AMOVA, samples clustered significantly between day 2 and month 6 (*P* < .003) and between month 2 and month 6 (*P* < .003) but not between day 2 and month 2 (*P* = .102). **C,** Mean theta similarity coefficients of comparisons within samples of each time point. Higher theta values signify greater similarity. *P < .05 and ***P < .001, Wilcoxon rank sum test. *Post hoc P* values were adjusted with the Bonferroni correction (n = 3). *Ch*, Cheeks; *PC*, principal component.

Skin microbiomes differ between children and adults; however, studies with longitudinal skin sampling in infants are infrequent.^27^, ^28^ Alterations in skin bacterial abundances at different sampling time points were apparent in our cohort (see Fig E4 in this article's Online Repository at www.jacionline.org). For each skin site, the bacterial community structures showed striking shifts based on the sampling time point (Fig 2, *A* and *B*, and see Fig E5 in this article's Online Repository at www.jacionline.org). At both extremity sites, the samples clustered separately between day 2 and month 6 (*P* = .024 for Af and *P* = .003 for Pf, AMOVA). For both facial sites, day 2 and month 6 samples clustered significantly (*P* < .003 for each), as did those between month 2 and month 6 (*P* < .003 at the cheeks and *P* = .06 at Nt).

To examine interpersonal variation, we calculated the mean similarity between samples at a single site and time point. For both facial sites, bacterial communities between subjects were most similar at month 6, converging to a more common bacterial population across subjects. Extremity sites did not present this same pattern; instead, the most similar bacterial community structures were observed at month 2 (Fig 2, *C*).

**Fig 3:**
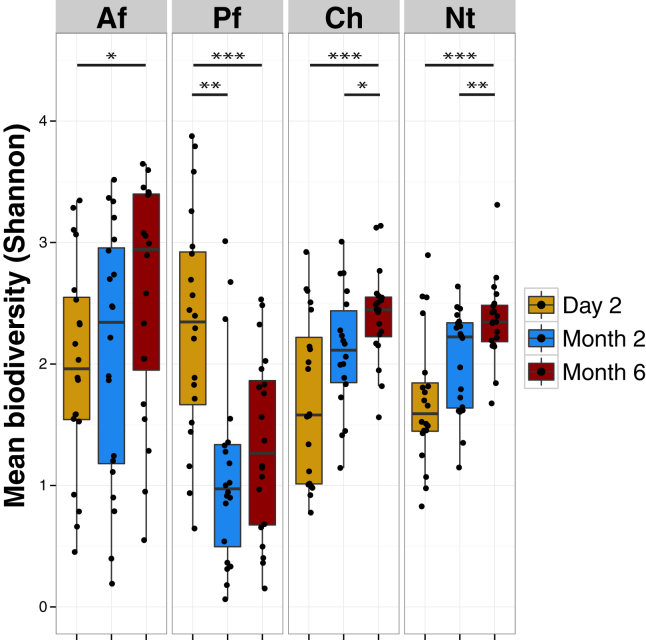
Changes in bacterial biodiversity with age. Mean Shannon diversity, calculated based on the richness and evenness of taxa within the community, at each time point is shown. *P < .05, **P < .01, and ***P < .001. *Ch*, Cheeks; *PC*, principal component.

We analyzed the bacterial diversity of all samples by using the Shannon diversity index (a higher value signifies more taxonomic groups, a more even distribution of these groups, or both). At each time point, diversity was similar between Af, cheeks, and Nt (*P* > .05, Wilcoxon rank sum test; Fig 3 and see Table E3 in this article's Online Repository at www.jacionline.org). Pf had a substantially altered pattern, significantly different from the other sites at all time points, except Af at day 2. At the facial sites, bacterial diversity increased significantly over the time studied (*P* < .001 for each site between day 2 and month 6, Wilcoxon rank sum test; see Table E3). Combined with the increasingly similar bacterial community structures on the face, this suggests that over time, the microbial population converges and stabilizes at facial sites. Samples from the Af also significantly increased in diversity between the time points (*P* = .033).

### Colonization of Af with commensal staphylococci at month 2 is associated with decreased incidence of AD at 1 year

To identify any bacterial differences associated with AD in this cohort, we compared infants with AD at any time within the first year of life versus control subjects for each site and sampling time. At all time points, the bacterial community structures of infants with AD at any time within the first year of life did not cluster separately from control infants, and no significant differences in Shannon diversity were identified between the groups. Because the patients had clinical disease presenting at different time points (see Table E1), we also compared samples based on whether the subjects presented with disease at each time point. Overall, there was almost no distinction between affected and unaffected samples in within- or between-sample diversity, either before or at the time point when the patients were affected (see Tables E3 and E4 in this article's Online Repository at www.jacionline.org).

**Fig 4:**
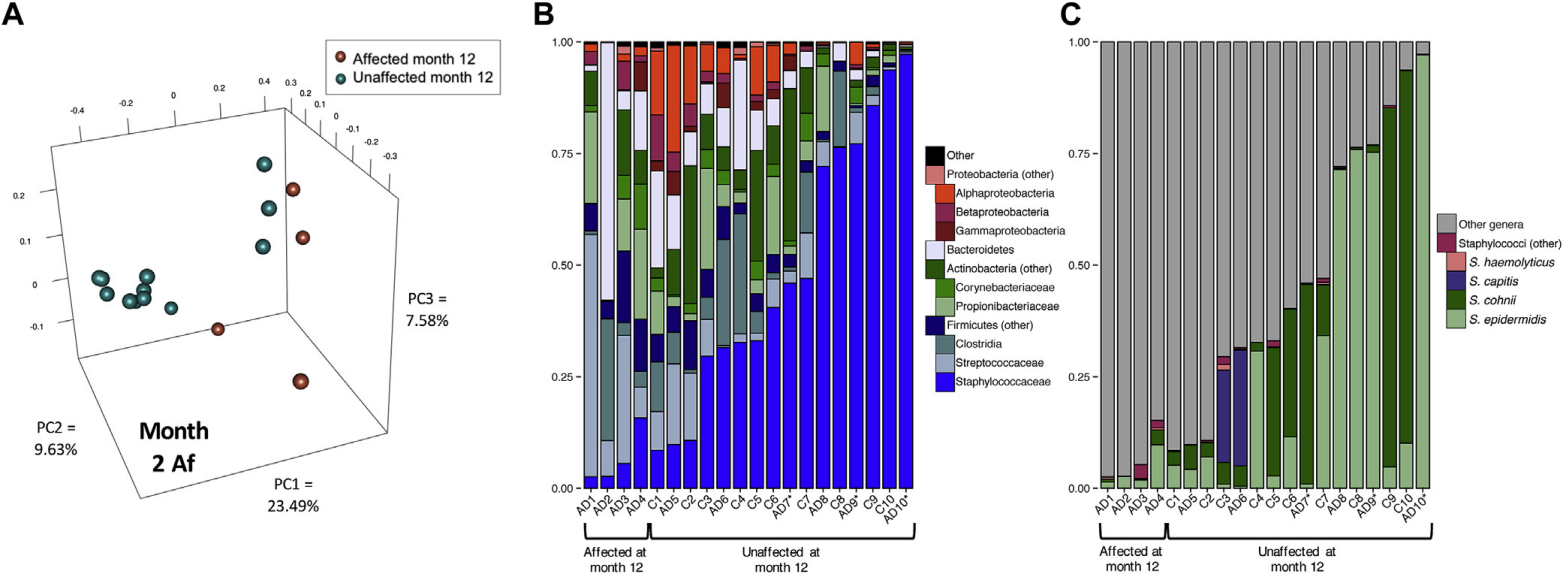
Af microbial community differences predate AD presentation. **A,** Month 2 Af samples were examined by using principal coordinates analysis of the theta similarity coefficient. Samples were clustered by those that went on to be affected at month 12 and those that were unaffected at month 12 (*P* = .003, AMOVA). **B,** Relative abundance of major taxa. Subjects who went on to be affected at month 12 had significantly lower proportions of *Staphylococcus* species than those who went on to be unaffected (*P* = .008, Wilcoxon rank sum test). **C,** Relative abundance of staphylococcal species. *S aureus* was essentially absent in these communities. *PC*, Principal component.

Interestingly, the month 2 Af samples demonstrated statistically significant clustering grouped by those infants who went on to be affected at month 12 in this study (*P* = .003, AMOVA). OTU-based analysis suggested that a single OTU was differentially abundant between the groups; this OTU was classified as *Staphylococcus* (Fig 4, *A*). When considering all sequences classified to the *Staphylococcus* genus, the relative abundances were significantly different between the 2 groups, with subjects who went on to be affected colonized by significantly less staphylococci (mean, 0.065; 95% CI, 0.035-0.094) compared with those who went on to be unaffected (mean, 0.495; 95% CI, 0.458-0.531; *P* < .003, Wilcoxon rank sum test; Fig 4, *B*).

**Fig E6:**
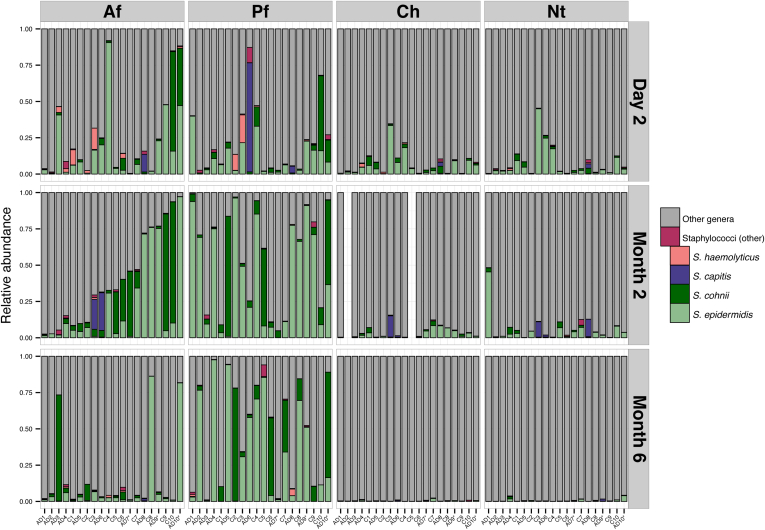


**Fig E7:**
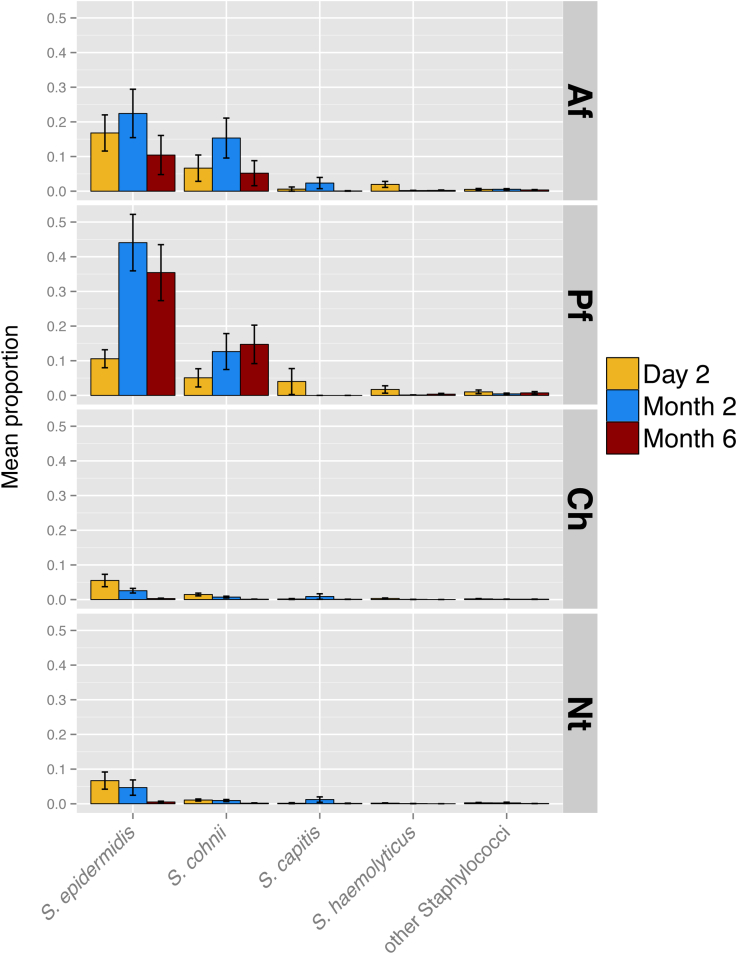


Given the specific association between *S aureus* and AD flares, we classified the *Staphylococcus* sequences to the species level; in these samples the most prevalent species were *Staphylococcus epidermidis* and *Staphylococcus cohnii* (Fig 4, *C*). In contrast to older patients with AD or patients with more severe AD,^29^ essentially no *S aureus* sequences were present in the samples in our cohort, even at the sites and times that patients were affected (see Figs E6 and E7 in this article's Online Repository at www.jacionline.org). There were no statistically significant differences within individual *Staphylococcus* species levels in the month 2 Af samples between the later-affected and later-unaffected samples.

### Birth method and feeding method have little effect on skin microbiota

**Fig E8:**
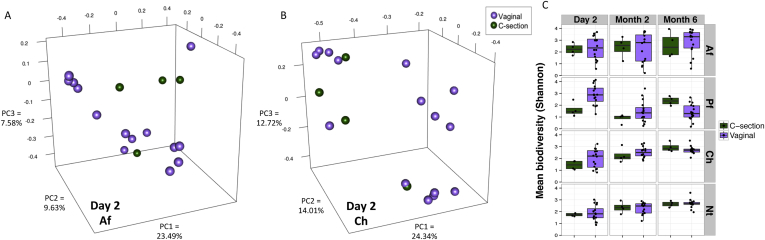


Differences have previously been reported between the skin microbiota of infants born by means of cesarean section versus vaginal birth.^28^ We investigated whether birth method was associated with differences in the skin microbiota in our cohort. There was no clustering of samples at any site or time point based on birth method, except Af samples at day 2 (see Fig E8, *A* and *B*, in this article's Online Repository at www.jacionline.org). Shannon diversity was similar between the 2 birth methods as well (see Fig E8, *C*). Feeding method has been associated with differences in the intestinal microbiome composition of infants.^30^, ^31^ However, feeding method (breast, formula, or combination) and sex did not affect skin bacterial colonization patterns in this cohort (see Tables E3 and E4).

## Discussion

Although infections with *S aureus* and herpes simplex virus can complicate the course of established AD, the role of microbes in the cause, genesis, and pathogenesis of AD remains unclear. Recent murine studies have shown that cutaneous microbes can influence the development of skin immunity and disease.^5^, ^6^, ^9^ Determining whether cutaneous microbes play a role in the initiation of AD could provide an opportunity to reduce the development of atopic disorders. To investigate the skin microbiome in infants before the development of AD, we used 16S rRNA gene sequencing of skin samples from a birth cohort and determined that shifts occur in the skin microbiome over the first 6 months of life, with site-specific bacterial communities changing in composition and diversity over time. We also identified a difference in staphylococcal colonization at a site of AD predilection that predates the presentation of disease, with patients who went on to be affected at a later date colonized by fewer *Staphylococcus* species. Birth method and feeding method did not appear to affect skin bacterial communities at the sites and time points studied in this cohort, but other studies are needed to confirm these findings.

Prior studies of human skin have shown that skin microbial communities are site specific.^32^, ^33^ Although there is heterogeneity of bacterial communities across the skin surface, specific skin sites in different subjects often share common patterns of bacterial composition. This biogeography of the skin microbiome has been observed in older children and adults.^26^, ^34^ In previous infant skin microbiome studies, site-specific differences were not evident in the first 3 months of life because infants were studied at a single time point immediately after delivery or were sampled at a single time point and analyzed in age cohorts of 1 to 3, 4 to 6, and 6 to 12 months.^27^, ^28^ The present study differed by sampling the same cohort of infants over a 6-month interval (day 2, month 2, and month 6) and observed site-specific differences as early as the second day of life, a time point not previously investigated. The bacterial diversity of 1 skin site, the Pf, shifted at time points differentially from the 3 other sites studied. Because this specific skin site has not been examined in a cohort this young, the results might be related to a unique aspect of infant skin physiology, exposure, or both or specific to this cohort. Interestingly, the body site differences in bacterial communities also reflect observed site differences in immune cell density and composition from human skin.^35^, ^36^, ^37^, ^38^ Investigating site-specific differences in host-microbial interactions can enhance our understanding of the predilection of certain skin regions for dermatologic diseases.

In addition to the biogeography of the skin microbiome, skin bacterial communities can shift significantly during different periods of the lifecycle, such as puberty.^26^ The physiology of infant skin changes over the first year of life, with alterations in stratum corneum hydration, skin pH, and sebum production.^39^ In this study the shifts in skin bacterial communities in the first months of life were the inverse of skin microbiome alterations that have been observed later in childhood. The increasing Shannon diversity observed in the first year of life in this infant cohort supports previous work that showed increased evenness or similar numbers in each taxa in bacterial communities from 3 skin sites in a cross-sectional study.^27^ During puberty, significant shifts in skin bacterial communities likely reflect the changes in skin physiology and systemic hormones.^26^ Changes in the skin microbiome observed in these infants potentially reflect the influences of waning maternal hormones, as well as the continued development of infant skin. For example, lipophilic *Propionibacterium* species are relatively abundant on the facial sites at neonatal day 2 but decrease substantially at later time points. This corresponds to the high sebaceous activity triggered by maternal hormones in the first days of life, which wane significantly in the weeks after birth.^40^ These findings lead to additional questions, including whether neonatal skin disorders, such as cephalic pustulosis (also known as neonatal acne), attributed to maternal hormones potentially might also be affected by alterations in skin bacteria.

A previous study in mice reported that having antigen-specific tolerance to commensals depends on early colonization, suggesting that there is a “critical window” for inducing regulatory T cells, which prevent a later inflammatory response to these bacteria.^6^ Scharschmidt et al^6^ showed that application of a commensal species of *Staphylococcus* on neonatal skin induced these immunomodulatory effects. The relatively low abundance of pathogenic staphylococcal species on the Af of 2-month-old infants who later had AD at 12 months of age is intriguing in the context of this prior work in mice. Whether cutaneous exposure to commensal staphylococci during early infancy can have a similar effect remains unknown, and further investigation is needed to understand whether this might influence the development of AD. The absence of *S aureus* in AD lesions of this cohort was somewhat surprising, given that this species is associated with AD.^29^, ^41^, ^42^, ^43^ A culture-based analysis of infant skin demonstrated *S aureus* colonization in approximately 21% of AD lesions among infants in their first year of life.^44^ The differences might be related to inherent differences in the study populations and/or the severity of sampled skin lesions between the study groups.

Differences in birth method have been studied in relation to the incidence of atopy and the neonatal skin microbiome.^28^, ^45^ An earlier study showed skin microbiome differences based on birth method in neonates sampled a few minutes after delivery. The small sample size and rare number of cesarean deliveries in the current study potentially contribute to the lack of statistically significant differences between the skin microbiota of infants born vaginally or by means of cesarean section at the earliest time point in this study, day 2 of life. Although this study analyzed different sites over a longer timeframe than the previous work, a larger study would be needed to address this question. Birth method might determine skin colonization very early in life; however, environmental exposures and skin physiology might predominate in shaping bacterial communities after this initial delivery. The skin barrier and *FLG* mutations are additional aspects of skin physiology that have been studied in relation to atopy. Although approximately 10% of subjects in BASELINE publications and the Irish population have *FLG* mutations, the current cohort had fewer *FLG* mutations than expected because of sampling effects. Because a large proportion of patients with AD do not carry *FLG*-null alleles, the results in the current cohort avoid the potential effects of *FLG* mutations and remain relevant to AD. With interest in the potential immunologic effects of neonatal exposures to skin microbes,^6^, ^46^ characterizing the early skin microbiome in neonates with and without *FLG* mutations and the timeframe for possible development of immune tolerance would be of significant clinical importance.

There are increasing efforts to understand the potential relationship between the skin microbial landscape and the development of skin immunity and human disease. Early studies of the skin microbiome will identify possible associations between specific microbes and human health and disease, but extensive further research will be required to unravel the pathophysiology and key mechanisms involved. Longitudinal sampling of the same subjects as internal controls and the initiation of sampling soon after birth were features of this study that improve the ability to identify distinct microbial patterns that could provide insight into the skin microbial milieu before the development of skin disease. As a result, we were able to define the site specificity and longitudinal shifts of the skin microbiome in the first 6 months of life, as well as the difference in relative abundances of commensal staphylococci before the development of AD. Additional investigations are needed to test whether site-specific differences in skin microbes influence the development of AD.

**Clinical implications**

*S aureus* colonization was absent in infants with AD. Colonization by commensal staphylococcal species might protect against eczema.

## Figures and Tables

**Table I:** Demographic data for study subjects

	Healthy control subjects	Patients with AD
Female/male sex	6/4	5/5
Cesarean section/vaginal delivery	1/9	3/7
BF/FF/C	2/3/5	1/2/7
Rural/urban	5/5	4/6
Pet/no pet	5/5	3/7
Emollient use (month 2), yes/no	2/8	6/4
Bathing frequency (month 2), ≤ weekly/> weekly	5/5	6/4
Antibiotic use (month 2), yes/no	1/9	1/9
Antibiotic use (6 mo), yes/no	5/5	3/7
TEWL		
Day 2	9.668 ± 0.776	9.749 ± 0.618
Month 2	10.402 ± 1.619	11.124 ± 2.135
Month 6	11.412 ± 2.149	10.08 ± 1.342

*BF*, Breast-fed exclusively; *C*, combination feeding; *FF*, formula fed exclusively; *TEWL*, transepidermal water loss.

## Supplementary data

Tables E1–E4

Legends for Figs E1-E8
