# Supplementary material for: Skin microbiome before development of atopic dermatitis: Early colonization with commensal staphylococci at 2 months is associated with a lower risk of atopic dermatitis at 1 year
Source: J Allergy Clin Immunol. 2017 Jan;139(1):166–72. doi: 10.1016/j.jaci.2016.07.029 (PMC5207796; doi:10.1016/j.jaci.2016.07.029)
Supplement: Legends for Figs E1-E8 [file mmc2.docx]

**Fig E1**

Rarefaction curves for sampling at each site to a cutoff of 1000 sequences; OTUs calculated at a cutoff of 97% nucleotide similarity. Each point represents mean ± SEM for all subjects at the site and sampling time indicated.

**Fig E2**

Relative abundance of major taxa; each bar represents a subject sampled at a single site and time point.

**Fig E3**

All samples month two and month six clustered by principal coordinates analysis based on theta similarity coefficients. At month two, Af and Pf had similar centroids (AMOVA p-value=0.18), as did Ch and Nt (p=0.276), and each still clustered distinctly from the other site pair (p < 0.006). At month six, Af and Pf had distinct centroids (AMOVA p=<0.006), but Ch and Nt clustered together (p=1); and the two site pairs clustered distinctly (p < 0.006).

Post-hoc p-values adjusted with Bonferonni correction (n=6)

**Fig E4**

Mean of relative abundance of major taxa; each bar represents the mean ± SEM of all subjects at a single site and time point.

**Fig E5**

1. Popliteal fossa samples clustered by principal coordinates analysis based on theta similarity coefficients. Using AMOVA, samples clustered significantly between day two and month six (p = 0.003), between day two and month six (p = 0.042), but not between month two and month 6 (p=1).
2. Nasal tip samples clustered by principal coordinates analysis based on theta similarity coefficients. Using AMOVA, samples clustered distinctly between day two and month two (p=<0.003), between day two and month six(p=<0.003), and between month two and month six (p=0.06).

Post-hoc p-values adjusted with Bonferonni correction (n=3)

**Fig E6**

Relative abundance of Staphylococcal species; each bar represents a subject sampled at a single site and time point.

**Fig E7**

Mean of relative abundance of Staphylococcal species; each bar represents the mean ± SEM of all subjects at a single site and time point.

**Fig E8**

1. Day 2 antecubital fossa samples clustered by principal coordinates analysis based on theta similarity coefficients. Samples clustered by birth method (AMOVA p-value = 0.005)
2. Day 2 cheek samples by principal coordinates analysis of theta similarity coefficient. Samples did not cluster by birth method (AMOVA p-value = 0.337).
3. Shannon diversity was generally similar between the birth methods, with only the popliteal fossa significantly different at day two (Wilcox rank-sum test p-value = 0.016)
